# DSOF: A Rapid Method to Determine the Abundance of Microalgae and Methanotrophic Bacteria in Coculture Using a Combination of Differential Sedimentation, Optical Density, and Fluorescence

**DOI:** 10.3390/bioengineering12091000

**Published:** 2025-09-19

**Authors:** Carlos Cartin-Caballero, Christophe Collet, Daniel Gapes, Peter A. Gostomski, Matthew B. Stott, Carlo R. Carere

**Affiliations:** 1 Te Tari Pūhanga Tukanga Matū | Department of Chemical and Process Engineering, Te Whare Wānanga o Waitaha | University of Canterbury, Christchurch 8140, Aotearoa-New Zealand; 2 Scion, Te Papa Tipu Innovation Park, Rotorua 3010, Aotearoa-New Zealand; 3 CIIBio, Universidad Nacional, Heredia 86-3000, Costa Rica; 4 Cetogenix, c/o Scion, Private Bag 3020, Rotorua 3046, New Zealand; 5 Te Kura Pūtaiao Koiora-School of Biological Sciences, Te Whare Wānanga o Waitaha-University of Canterbury, Christchurch 8140, Aotearoa-New Zealand

**Keywords:** microalgae, methanotroph, coculture, bioprocess, extremophile, single cell protein, bioprocess monitoring, coculture quantification

## Abstract

Cocultivation of microalgae and aerobic methanotrophs represents an emerging biotechnology platform to produce high-protein biomass, yet quantifying individual species in mixed cultures remains challenging. Here, we present a rapid, low-cost method—differential sedimentation, optical density, and fluorescence (DSOF)—to determine the abundance of coculture members. DSOF exploits differences in cell size and pigment autofluorescence between the thermoacidophilic microalga and methanotrophic species *Galdieria* sp. RTK37.1 and *Methylacidiphilum* sp. RTK17.1, respectively, to selectively sediment algal cells and estimate population contributions via OD_600_ and phycocyanin fluorescence. Evaluation with model suspensions across a wide cell density range (0 ≤ [*Galdieria*]: ≤ 3.23 A.U., and 0 ≤ [*Methylacidiphilum*] ≤ 1.54 A.U.) showed strong agreement with known values, with most absolute errors < 0.1 A.U. and relative errors < 10% at moderate biomass levels. Application to live batch cocultures under microalga or methanotroph growth-suppressed conditions, and during simultaneous growth, demonstrated accurate tracking of population dynamics and revealed enhanced methanotroph growth in the presence of oxygenic microalgae. While DSOF accuracy decreases at very concentrated biomass (>2.0 A.U. for *Galdieria*) or under nitrogen-limiting conditions, the model provides a practical, scalable alternative to more complex, invasive or expensive techniques, enabling near real-time monitoring of microalgae–methanotroph cocultures.

## 1. Introduction

The cocultivation of microalgae and aerobic methane-oxidizing bacteria (methanotrophs) presents a promising strategy for sustainable biotechnology applications, particularly in the development of high-protein biomass suitable for animal feed [1,2,3]. These microbial partnerships leverage complementary metabolisms (e.g., oxygen production and consumption), creating synergistic interactions under specific environmental conditions [1]. Thermoacidophilic microalgae–methanotroph cocultures further offer a robust and resilient platform for single cell protein (SCP) production, as their ability to thrive in extreme conditions limits contamination and supports continuous cultivation [4,5]. Recently, cocultures of the microalga *Galdieria* sp. RTK37.1 and the methanotroph *Methylacidiphilum* sp. RTK17.1 produced biomass with nutritional quality comparable to soybean meal and fishmeal, highlighting their potential as a high-protein feed for animals [6]. Beyond protein yield, such cocultures align with circular bioeconomy principles, enabling methane bioconversion into SCP and other products, while mitigating greenhouse gas emissions and integrating into wastewater valorisation systems [7].

Differentiating the relative proportions of each microorganism in coculture is critical for optimizing protein-enriched biomass production and ensuring consistent process control. *Galdieria* spp. have been reported to exhibit high protein content—ranging between ~26 and 68% (*w*/*w*) and contain a significant fraction of essential amino acids (~43%) [6,8,9]. Similarly, methanotrophic or methylotrophic bacteria can exhibit protein levels approximating 50–70% of dry biomass [6,10]. In both algal–bacterial systems and applied settings such as wastewater treatment, inoculum ratios strongly influence biomass yield, protein/carbohydrate/lipid composition, and granule stability, all of which depend on accurate differentiation of algal and bacterial fractions [3,11,12,13,14]. Accurate quantification of coculture members is therefore essential not only for studying microbial interactions but also to maximize productivity, reproducibility, and nutritional quality in industrial-scale SCP production.

Microalgae–methanotroph coculture dynamics are often reported as a total biomass concentration (i.e., optical density, dry biomass) without differentiating the relative proportion of each microorganism [1,6]. When relative abundance is reported, flow cytometry is typically used for direct cell counting [3,15]. Rarely, different techniques are combined to quantify coculture dynamics (e.g., flow cytometry in concert with selective plate counting) [16]. Cell counting via flow cytometry is typically fast, with low instrumental error (<5%) and high sensitivity [17]. However, it requires dedicated, expensive equipment and highly trained technical support [18,19]. In addition, cell fixation is sometimes required to preserve samples or to make cells permeable to dyes [20]. If protocols are not optimized, substantial count errors can result [21]. Other enumeration methods, such as calculating biomass concentrations using direct cell counts, can be problematic as biomass accumulation can occur in the absence of cell division via glycogen [22,23,24] or polyhydroxybutyrate (PHB) accumulation [25]. More recently, non-invasive monitoring tools such as online fluorescence probes and imaging-based sensors have emerged, but they lack taxonomic specificity in mixed cultures [26,27]. Broader bioprocess monitoring technologies, including software sensors, machine learning algorithms, and multi-spectral probes, have advanced in photobioreactor systems, yet their application to complex cocultures remains underdeveloped [26,28,29]. Monitoring mixed microalgae–bacteria systems remains a major challenge, as physical and biological traits (i.e., cell aggregation, sedimentation, and pigment content) influence biomass dynamics but are difficult to disentangle with conventional methods [30]. This highlights the need for rapid, scalable tools to differentiate microbial groups within consortia. Existing techniques, such as flow cytometry and qPCR, though powerful, are often too costly and resource-intensive for routine use. To scale up coculture-based SCP production, simple, rapid, and inexpensive alternatives are required to replace these invasive and high-cost methods.

Computational methods can quantify the proportional biomass of photoautotroph-methanotroph coculture members by combining mass-balance approaches with individual growth yield coefficients; for instance, the E-C method [21] combines total optical density (with wavelength dependent on the coculture pair), headspace gas composition (e.g., CH_4_, CO_2,_ and O_2_), and dissolved CO_2_ concentrations. Unfortunately, its reliance on monitoring gas concentration dynamics makes it impractical in small-volume reactors, as repeated headspace samplings are likely to impact experimental conditions. Additionally, changes to headspace pressure or gas flow rates could cause significant errors if not properly accounted. Similar methods, which inject an inert gas (e.g., Ar) to compensate for pressure loss or serve as a tracer, have key limitations as they can: (1) alter headspace composition, (2) interfere with GC analyses due to common carrier gases (Ar, N_2_, He), and (3) fail when cocultures generate more gas than they consume [31]. Finally, mass balance-based methods for disambiguating coculture member species require precise knowledge of yield coefficients and often assume their constancy, which is rarely the case. For instance, in *Scenedesmus obliquus*–*Methylocystis bryophila* cocultures, biomass yields increased from 0.18 to 0.30 mol_C_ mol_CH4_^−1^ as O_2_ increased from 10 to 50% *v*/*v* [32]. Similar variability is likely with *Methylacidiphilum* spp., which can utilize metabolites from partner species (e.g., methanol, H_2_/CO_2_, formate), altering CH_4_-to-biomass yields [23,33,34].

Nevertheless, the physical characteristics of microorganisms (e.g., cell size, shape, density, and fluorescence) can aid in estimating the relative abundance of species within a coculture. Optical density (OD) is commonly used to correlate biomass concentration in axenic cultures [31], with specific wavelengths employed to quantify *Galdieria* spp. (e.g., 750 and 800 nm) [22,35,36] and *Methylacidiphilum* spp. (e.g., 600 nm) [33,37]. However, OD at a single wavelength does not produce unique biomass profiles in mixed cultures [31]. Microalgae possess photosynthetic pigments (e.g., chlorophyll, phycocyanin, and carotenoids) that exhibit autofluorescence and are often measured in vivo to estimate pigment concentration [38,39,40]. For example, phycocyanin in *Galdieria* fluoresces at 670 nm when excited at 590 nm. While pigment content can approximate photosynthetic biomass [41], it fluctuates with cell physiology, age, light quality, and growth conditions, limiting its reliability for tracking algal growth [38,42]. Recent studies have explored integrating spectroscopic approaches, combining optical density and fluorescence to improve algal biomass estimation [43]. These methods demonstrate the value of leveraging multiple optical signals for rapid and low-cost quantification in monocultures, yet they remain largely untested in mixed microbial systems where overlapping signals complicate analysis.

In this study, we aimed to design and evaluate a simple, low-cost method to quantify the relative abundance of thermoacidophilic microalgae and methanotrophic bacteria in cocultures by combining differential sedimentation, optical density, and autofluorescence (hereafter referred to as DSOF). Unlike flow cytometry or mass-balance models, the proposed method relies on easily measurable physical properties, such as cell size and pigment fluorescence, allowing quantification of coculture members with minimal equipment. Using this approach, the individual contributions of *Galdieria* sp. RTK37.1 and *Methylacidiphilum* sp. RTK17.1 were obtained across a broad range of biomass concentrations with acceptable error. Collectively, these findings demonstrate that the DSOF method provides a practical, low-cost, and scalable approach for quantifying coculture member dynamics, offering a valuable alternative to more complex or invasive techniques for resolving species abundance in microalgae–methanotroph systems.

## 2. Materials and Methods

**Figure 1:**
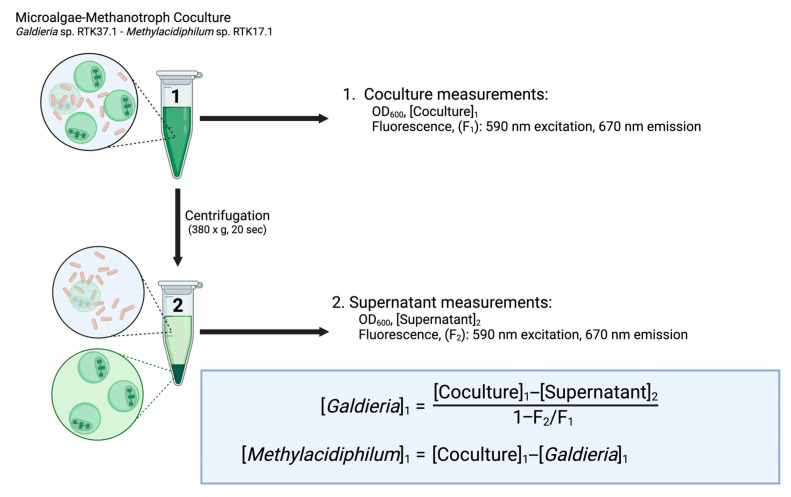
Graphical summary of the differential sedimentation optical density/fluorescence (DSOF) method. Image produced using BioRender.

The DSOF method was developed to exploit differences in the settling velocity and autofluorescence of a microalga (*Galdieria* sp. RTK37.1) and a methanotrophic bacterium (*Methylacidiphilum* sp. RTK17.1) and to determine the concentration of each microorganism in coculture. As a conceptual overview, a small volume of coculture is harvested and briefly centrifuged under weak centrifugal forces. Supernatant fluorescence and optical density are then compared to those obtained from the uncentrifuged sample. With these four values, the optical density (and by correlation, biomass concentration) of the bacterium (*Methylacidiphilum* sp. RTK17.1) and microalga (*Galdieria* sp. RTK37.1) in the original coculture can be calculated (Figure 1).

The basis of the DSOF method is to exploit the cell size difference between species: *Galdieria sulphuraria* cells range between ~3 and 9 µm in diameter [44], whereas *Methylacidiphilum* spp. are smaller rods (0.8–2.0 µm length, 0.40–0.65 µm width) [45,46]. According to Stokes’ law, sedimentation velocity scales with the square of particle diameter [47], suggesting *Galdieria* cells settle ~100× faster than *Methylacidiphilum*. Therefore, gentle centrifugation (<400× *g*) could selectively pellet microalgal cells while leaving bacterial cells in suspension. Measuring the change in OD and pigment fluorescence in the resulting supernatant could then be used to estimate the quantity of separated microalgae—and by extension, their abundance in the original sample.

**Figure A1:**
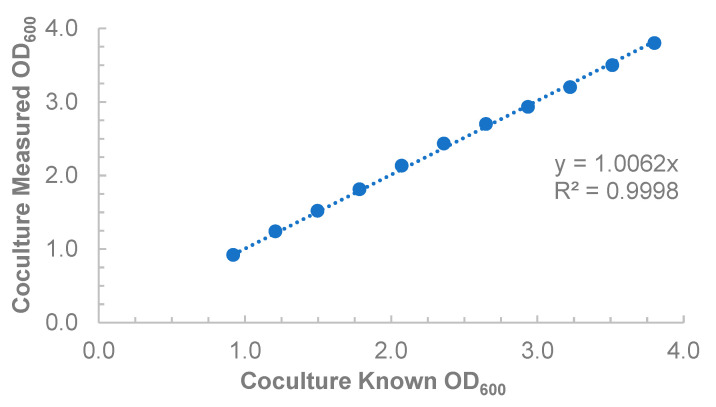
Comparison of model “coculture suspension” OD_600_ with the summation of *Methylacidiphilum* sp. RTK17.1 and *Galdieria* sp. RTK37.1 individual OD_600_ values. The resulting linear regression model and coefficient of determination (R^2^) are shown.

**Figure A2:**
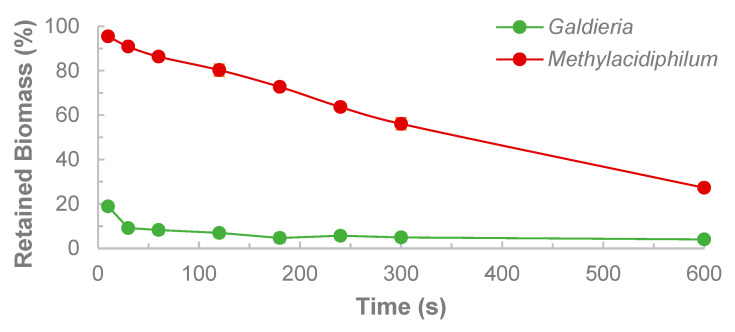
Fraction of retained biomass in the supernatant for *Methylacidiphilum* sp. RTK17.1 (OD_600_ = 0.88) and *Galdieria* sp. RTK37.1 (OD_600_ = 2.4) suspensions.

The DSOF method assumes that at 600 nm, the absorbance of *Methylacidiphilum* sp. RTK17.1 and *Galdieria* sp. RTK37.1 is additive (Figure A1), so for any given coculture sample:

Supposition 1: OD_600_ values are additive for *Methylacidiphilum* sp. RTK17.1 and *Galdieria* sp. RTK37.1, then:

(1) $${\left[Methylacidiphilum\right]}_{1}+{\left[Galdieria\right]}_{1}={\left[Coculture\right]}_{1}$$

where subscript “1” denotes measurements made to the uncentrifuged coculture sample. Due to their larger cell size, *Galdieria* sp. RTK37.1 exhibits significantly faster settling velocities than *Methylacidiphilum* sp. RTK17.1. Therefore, brief centrifugation at low centrifugal force is expected to selectively pellet the microalgal cells while leaving most methanotrophs suspended. As cells settle out of suspension, the optical density of the coculture decreases; thus, the OD of the supernatant after centrifugation reflects the remaining, predominantly bacterial, biomass:

(2) $${\left[Methylacidiphilum\right]}_{2}+{\left[Galdieria\right]}_{2}={\left[Supernatant\right]}_{2}$$

where subscript “2” refers to measurements made to the supernatant resulting from coculture centrifugation. The second assumption for the DSOF method is that there is no significant sedimentation of methanotrophic cells:

Supposition 2: There is no significant sedimentation of methanotroph cells (Figure A2), then:

(3) $${\left[Methylacidiphilum\right]}_{1}={\left[Methylacidiphilum\right]}_{2}$$

*Galdieria* sp. RTK37.1 cells are partially removed by centrifugation, so in the supernatant, only a fraction of microalgae cells, “*y*”, remains, then the following is written:

(4) $${\left[Galdieria\right]}_{2}=y\cdot {\left[Galdieria\right]}_{1}$$

**Figure A3:**
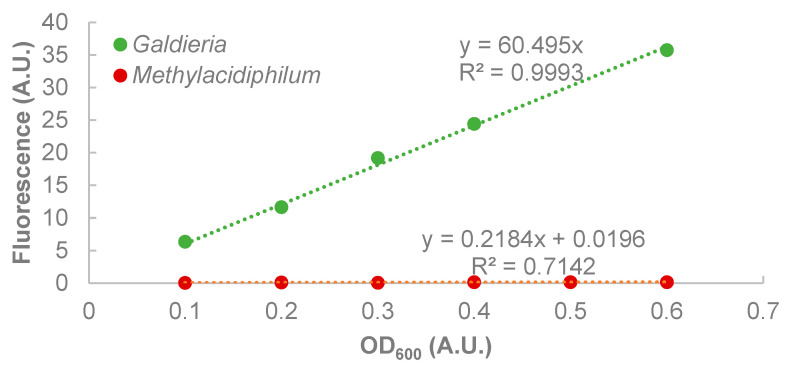
Fluorescence (670 nm emission, 590 nm excitation) as a function of OD_600_ for suspensions of *Methylacidiphilum* sp. RTK17.1 and *Galdieria* sp. RTK37.1. The resulting linear regression model and coefficient of determination (R^2^) are shown.

**Figure A4:**
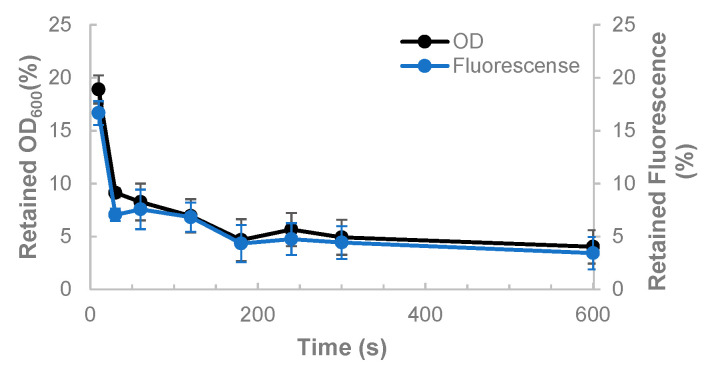
Fraction of retained OD_600_ and fluorescence (670 nm emission, 590 nm excitation) in the supernatant for suspensions of *Galdieria* sp. RTK37.

Phycocyanin, a pigment produced by *Galdieria* spp., exhibits fluorescence at 670 nm when excited at 590 nm [40]. If we assume *Methylacidiphilum* sp. RTK17.1 shows no fluorescence (Figure A3), and the fluorescence of phycocyanin is proportional to the microalgal cells remaining in suspension (Figure A4), which in turn is proportional to the optical density. Then, by measuring fluorescence of the original coculture (F_1_) and the supernatant (F_2_), we can approximate *y* as follows:

Supposition 3: The fraction of microalgae remaining in the supernatant can be approximated by the proportion of fluorescence of supernatant and original coculture as follows:

(5) y=Galdieria2Galdieria1=F2F1

Substituting Equations (4) and (3) in (2), to obtain the following system of equations:

(1) $${\left[Methylacidiphilum\right]}_{1}+{\left[Galdieria\right]}_{1}={\left[Coculture\right]}_{1}$$

(6) $${\left[Methylacidiphilum\right]}_{1}+y\cdot {\left[Galdieria\right]}_{1}={\left[Supernatant\right]}_{2}$$

Thus, we can solve for ${\left[Galdieria\right]}_{1}$:

(7) $${\left[Galdieria\right]}_{1}=\frac{{\left[Coculture\right]}_{1}-{\left[Supernatant\right]}_{2}}{1-y}$$

And for ${\left[Methylacidiphilum\right]}_{1}$:

(8) $${\left[Methylacidiphilum\right]}_{1}={\left[Coculture\right]}_{1}-\frac{{\left[Coculture\right]}_{1}-{\left[Supernatant\right]}_{2}}{1-y}$$

### 2.1. Technical Description of the DSOF Method

To differentiate the contribution of *Galdieria* sp. RTK37.1 and *Methylacidiphilum* sp. RTK17.1 to the coculture OD_600_, a 0.75 mL sample of coculture was harvested, and the optical density, [Coculture]_1_, was measured using an Ultrospec 10 cell density meter (Amersham Bioscience, Buckinghamshire, UK). Fresh V4 medium was used as a blank. If the sample OD_600_ was greater than 0.6, it was diluted in the modified V4 nutrient medium and remeasured. Next, 200 μL coculture was transferred into a black, flat-bottom (chimney well) 96-well microplate (Greiner Bio-One, Kremsmunster, Austria), and the fluorescence emission, F_1_, at 670 nm (590 nm excitation), was measured using a microplate reader (Varioskan Lux, Thermo Scientific, Waltham, MA, USA). Following this, a 1.5 mL coculture sample was transferred into a 1.5 mL Eppendorf tube and centrifuged at 380× *g* for 20 s (Biofuge Pico, Heraeus Instruments, Hanau, Germany). The resulting supernatant (0.75 mL) was then collected, and the OD_600_, [Supernatant]_2_, and fluorescence (F_2_) were measured as described for the coculture above. Finally, the OD_600_ values for *Galdieria* sp. RTK37.1 and *Methylacidiphilum* sp. RTK17.1 in coculture was then calculated using Equations (7) and (8), respectively.

### 2.2. Growth Medium and Culture Maintenance

Complete information on strain isolation and maintenance is provided in our previous work [6,22,33]. Briefly, both *Methylacidiphilum* sp. RTK17.1 and *Galdieria* sp. RTK37.1 were isolated from geothermally heated soils at Parariki in the Rotokawa geothermal area, Aotearoa-New Zealand [22,33]. Prior to experimentation, *Methylacidiphilum* sp. RTK17.1 was routinely maintained in chemostat culture at 50 °C and pH ~2.5 using a 1 L bioreactor (BioFlo 110; New Brunswick Scientific, Edison, NJ, USA, with 600 mL working volume, 0.0069 h^−1^ dilution rate, and agitation at 800 rpm) supplied with a CO_2_/CH_4_/O_2_/N_2_ gas mixture [33]. As previously described [6], *Galdieria* sp. RTK37.1 was propagated in batch cultures within sealed 1 L bottles containing V4 medium under 80% CO_2_/20% N_2_, incubated at 45 °C with continuous agitation and light. The bottles were incubated horizontally in a Lab Companion shaking incubator (Cole-Parmer, Chicago, IL, USA) at 110 rpm. Light was provided via three 50 W halogen lightbulbs and adjusted to 60 µmol_photons_ m^−2^ s^−1^. *Galdieria* cultures were routinely diluted to an OD_600_ of 1.0 at the beginning of each cycle and maintained until OD_600_ 6.0, at which point the next cycle was initiated.

A modified V4 medium [48] was used for all cultivation experiments. Briefly, the medium contained per liter: 0.4 g NH_4_Cl, 0.05 g KH_2_PO_4_, 0.02 g MgSO_4_·7H_2_O, 0.01 g CaCl_2_·2H_2_O, 3 mL FeEDTA solution, 3 mL trace elements solution, 1 mL trace metals solution, 0.2 µM Ce_2_(SO_4_)_3_, and 0.2 µM La_2_(SO_4_)_3_ solution and was adjusted to pH 2.5 with H_2_SO_4_. The FeEDTA solution was prepared by dissolving 1.54 g of FeSO_4_·7H_2_O and 2.06 g of Na_2_EDTA in one L of deionized water. The trace element solution was prepared by dissolving (per 1 L deionized water): 0.44 g ZnSO_4_.7 H_2_O, 0.20 g CuSO_4_.5 H_2_O, 0.19 g MnCl·4H_2_O, 0.06 g Na_2_MoO_4_.2 H_2_O, 0.10 g H_3_BO_3_, and 0.08 g CoCl_2_.6 H_2_O. To prepare the trace element solution, 1.5 g of Nitrilotriacetic acid was dissolved in 800 mL of deionized water and the pH was adjusted to 6.5 with KOH. Then the following minerals were dissolved in order: 0.2 g Fe(NH_4_)_2_(SO_4_)_2_·6H_2_O, 0.2 g Na_2_SeO_3_, 0.1 g CoCl_2_·6H_2_O, 0.1 g MnSO_4_·2H_2_O, 0.1 g Na_2_MoO_4_·2H_2_O, 0.1 g Na_2_WO_4_·2H_2_O, 0.1 g ZnSO_4_·7H_2_O, 0.04 g AlCl_3_·6H_2_O, 0.025 g NiCl_2_·6H_2_O, 0.01 g H_3_BO_3_, and 0.01 g CuSO_4_·5H_2_O. The pH was then adjusted to 7.0, and the volume brought to 1 L. Unless stated otherwise, all chemicals and reagents were purchased from Sigma-Aldrich (Darmstadt, Germany), and all gases were sourced from BOC (North Ryde, Australia).

### 2.3. Biomass Determination

Biomass concentrations were estimated from optical density at 600 nm (OD_600_) using previously established calibration curves [6]. For *Galdieria* sp. RTK37.1, 1.0 OD_600_ corresponded to 0.308 gDW L^−1^ in the range 0–9 OD_600_. For *Methylacidiphilum* sp. RTK17.1, 1.0 OD_600_ corresponded to 0.435 gDW L^−1^ in the range 0–2.5 OD_600_. These calibrations were used to convert OD_600_ measurements into biomass concentrations throughout this study.

### 2.4. DSOF Evaluation in Model Suspensions

To test the DSOF method, model “coculture” suspensions were prepared from axenic stock suspensions across a range of predefined OD_600_ values. For the methanotroph stock, the exponential phase of *Methylacidiphilum* sp. RTK17.1 cells were harvested from a 1 L bioreactor (BioFlo 110; New Brunswick Scientific, Edison, NJ, USA) grown in a chemostat (Dilution rate = 0.28 day^−1^) on a gas mixture of 67% CO_2_, 1.0% CH_4_, 2.1% O_2_ (balance N_2_, all *v*/*v*) supplied at 16 mL min^−1^. Temperature was kept at 45 °C, agitation at 400 rpm, and pH 2.5 (not controlled). Cells were stored at 4 °C until required, and then centrifuged (300 mL at a time in six 50-mL Eppendorf tubes) at 5000× *g* for 15 min in a 5810R Benchtop Centrifuge (Eppendorf, Hamburg, Germany, temperature controlled to 4 °C). Then, they were resuspended with sufficient V4 medium to achieve an OD_600_ of 4.0 A.U. For the microalgae stock, 300 mL V4 medium (pH 2.5) was inoculated with *Galdieria* sp. RTK37.1 to an initial OD_600_ of 1.0 A.U. within 1 L Duran Pressure Plus Bottles equipped with bromobutyl rubber stoppers. The bottles were then subjected to a vacuum for 3 min, and re-pressurized to 5 psi with an 80% CO_2_ and 20% N_2_ (*v*/*v*) gas mixture. The bottles were incubated in a shaker at 150 rpm and 45 °C. Illumination was supplied by warm white LED strips, providing 40 µmol photons m^−2^ s^−1^, measured at the outer surface of the bottle wall. Cells were harvested at OD_600_ = 3.5 A.U., which corresponds to the point at which NH_4_^+^ is depleted in the medium [6], to ensure cultures were never nitrogen-limited.

Several model coculture groups were prepared from the stock suspensions and analyzed with the DSOF to determine the method’s precision and accuracy. For each group, one of the microorganisms’ OD_600_ was kept constant, while the other was varied in defined increments. In total, eight groups were analyzed, five where the microalgae OD_600_ was constant ([*Galdieria*]_K_: 0.28, 0.52, 1.00, 1.81, and 3.23 A.U.), and three where the methanotroph OD_600_ was constant ([*Methylacidiphilum*]_K_: 0.44, 0.90, and 1.54 A.U.). For each suspension within each group, aliquots of both *Galdieria* sp. RTK37.1 and *Methylacidiphilum* sp. RTK17.1 was diluted into sterile V4 medium to the desired final optical density. Ten mL of each suspension was prepared. Triplicates of each model mixture were analyzed.

### 2.5. DSOF Evaluation in Live Batch Cocultures

To evaluate the DSOF method on growing cultures of *Galdieria* sp. RTK37.1 and *Methylacidiphilum* sp. RTK17.1, batch cocultures were performed under one of three conditions: (1) light, no CH4 to promote microalgae growth and suppress methanotroph growth, (2) no light, added CH4 to suppress microalgae growth and promote methanotroph growth, or (3) light, added CH4 to promote growth of both microorganisms. For these experiments, the previously described stocks of microalgae and methanotroph were used to inoculate 1 L Duran Pressure Plus Bottles, equipped with bromobutyl rubber stoppers, with 250 mL of broth in V4 medium (pH 2.5). The growth-suppressed cultures had a starting *Methylacidiphilum* sp. RTK17.1 OD_600_ value of 0.45 A.U., and a starting *Galdieria* sp. RTK37.1 value of 0.45 A.U. The non-suppressed cultures had initial OD_600_ values of 0.3 A.U. for *Methylacidiphilum* sp. RTK17.1, and 1.0 A.U. for *Galdieria* sp. RTK37.1. To promote methanotroph and suppress microalgal growth, bottles were covered with aluminum foil, sealed, and then 100 mL of CH_4_ and 60 mL of CO_2_ were injected. To promote microalgal growth and suppress methanotroph growth, bottles were injected with 160 mL of CO_2_, no CH_4_, and supplied with 40 µmol photons m^−2^ s^−1^ warm white LED lighting (i.e., no foil covering). To promote the growth of both methanotrophs and microalgae, bottles were injected with 100 mL of CO_2_, 60 mL of CH_4_, and illuminated as above. Table A1 shows the initial headspace gas concentrations for all conditions. The bottles were then cultivated on a shaking incubator (WiseCube WIS-10, Wisd Laboratory Instruments, Wertheim, Germany) at 45 °C and 150 rpm. Each condition was undertaken in triplicate.

Throughout the incubation period, 2 mL liquid samples were harvested daily and analyzed using the DSOF method. To ensure that CO_2_/CH_4_/O_2_ concentrations weren’t limiting growth, 20 mL headspace gas samples were analyzed daily using a 490 micro-GC equipped with a thermal conductivity detector (Agilent Technologies, Santa Clara, CA, USA). To replenish gases to their initial concentrations, after sampling, the bottles were opened on a Laminar Flow cabinet, left to equalize for 30 min, and re-gassed (Table A1). Growth was stopped after six days or when the total OD_600_ surpassed 3.5 A.U. Growth rates were calculated by fitting the appropriate concentration data to the exponential growth equation model in Prism Graphpad 9.4.1 ($X=X_{0}e^{\mu_{max}t}$, where X is the concentration in A.U., X_0_ is the concentration at time zero in A.U., µ_max_ is the specific growth rate in h^−1^, and t is time in hours). Comparison of growth rates (unpaired two-tailed *t*-tests, α = 0.05) was performed using Prism Graphpad 9.4.1.

## 3. Results and Discussion

### 3.1. Evaluation of the DSOF Method in Model Suspensions

**Figure 2:**
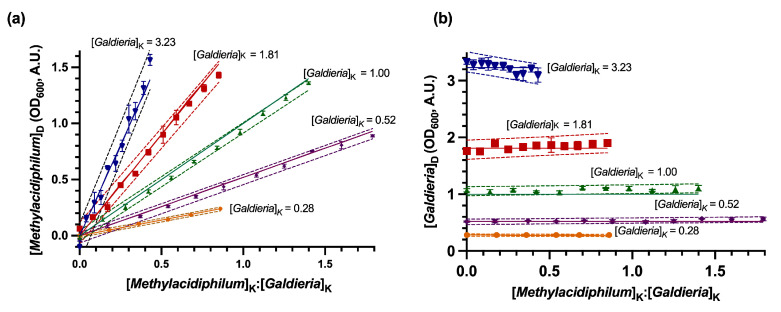
Derived concentrations of *Methylacidiphilum* sp. RTK17.1 and *Galdieria* sp. RTK37.1 in model (non-growing) suspensions using the DSOF method for the constant *Galdieria* concentration groups. (**a**) Derived *Methylacidiphilum* sp. RTK17.1 ([*Methylacidiphilum*]_D_) concentrations as a function of the known *Methylacidiphilum*:*Galdieria* concentration ratios in the model suspensions. (**b**) Derived *Galdieria* sp. RTK37.1 ([*Galdieria*]_D_) concentrations as a function of the known *Methylacidiphilum*:*Galdieria* concentration ratios in the model suspensions. Data points represent the average derived concentrations as determined by the DSOF method; error bars represent one standard deviation (*n* = 3). Dashed lines represent the limit of the 95% confidence prediction bands of the derived concentrations. Solid lines represent the linear regression (*y* = *mx* + *b*), where the known *Galdieria* concentration ([*Galdieria*]_K_) represents either the slope (panel a, *y* = *mx*) or the y-intercept (panel b, *y* = *b*). Model suspensions were made by dilution of the respective stocks. For each color group *Methylacidiphilum* sp. RTK17.1 concentrations were varied and *Galdieria* sp. RTK37.1 kept constant at the concentration (OD_600nm_) shown in graphs.

**Figure 3:**
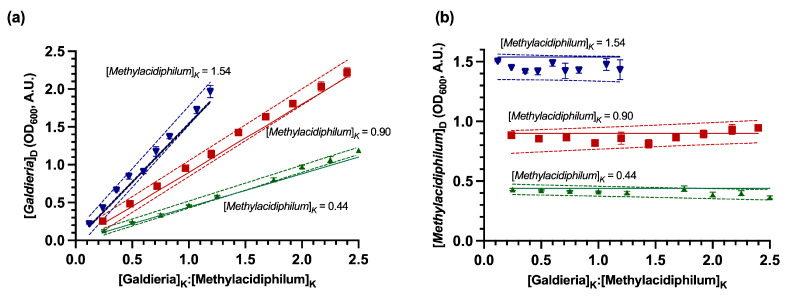
Derived concentrations of *Methylacidiphilum* sp. RTK17.1 and *Galdieria* sp. RTK37.1 in model suspensions using the DSOF method for the constant *Methylacidiphilum* concentration groups. (**a**) Derived *Galdieria* sp. RTK37.1 ([*Galdieria*]_D_) concentrations as a function of the known *Galdieria*:*Methylacidiphilum* concentration ratios in the model suspensions. (**b**) Derived *Methylacidiphilum* sp. RTK17.1 ([*Methylacidiphilum*]_D_) concentrations as a function of the known *Galdieria*:*Methylacidiphilum* concentration ratios in the model suspensions. Data points represent the derived concentrations as determined by the DSOF method; error bars represent one standard deviation (*n* = 3). Dashed lines represent the limit of the 95% confidence prediction bands of the derived concentrations. Solid lines represent the linear regression (y = mx + b), where the known *Methylacidiphilum* concentration ([*Methylacidiphilum*]_K_) represents either the slope (panel a, *y* = *mx*) or the y-intercept (panel b, *y* = *b*). Model cocultures were made by dilution of the respective stocks. For each color group, *Galdieria* sp. RTK37.1 concentrations were varied, and *Methylacidiphilum* sp. RTK17.1 remained constant at the concentration shown in the graphs.

Evaluation of the DSOF method in model (non-growing) suspensions confirmed good agreement between derived values (e.g., [*Galdieria*]_D_) from the DSOF method and known OD_600_ values of each microorganism (e.g., [*Galdieria*]_K_) in coculture (Figure 2). To assist with data visualization, known concentrations (e.g., [*Methylacidiphilum*]_K_) were normalized by dividing them by the corresponding concentration of the coculture partner (e.g., [*Galdieria*]_K_). Indicating a strong correlation, slopes of the derived concentration regressions were not significantly different from the slope of known concentration lines (as *y* = *mx*, with *m* = [*Galdieria*]_K_, *p*-value > 0.05, using a two-tailed unpaired *t*-test, Figure 2a). Likewise, the intercepts of derived regressions and the known concentration lines were not significantly different (Figure 2b, *p*-value > 0.05, using a two-tailed unpaired *t*-test). A similar analysis for the samples where *Methylacidiphilum* OD_600_ was kept constant along each group (Figure 3), shows no significant difference between the slopes of the derived concentration regressions and the slope of known concentration (*p*-value > 0.05 using a two-tailed unpaired *t*-test).

**Figure 4:**
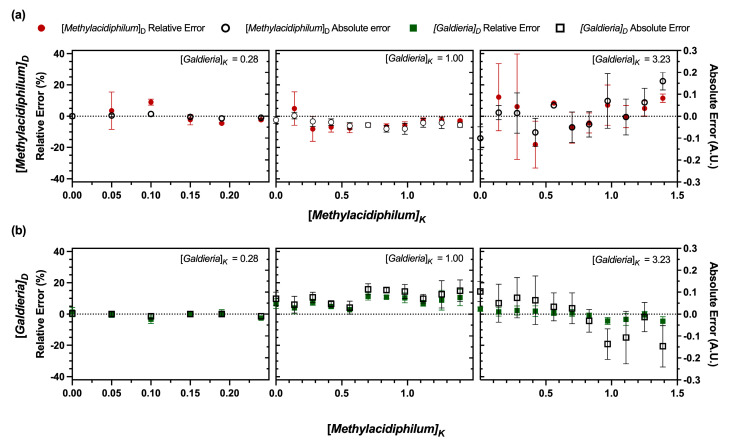
Relative and absolute errors of the derived concentrations of *Methylacidiphilum* sp. RTK17.1 and *Galdieria* sp. RTK37.1 in model suspensions using the DSOF method for the constant *Galdieria* concentration groups. (**a**) Derived *Methylacidiphilum* sp. RTK17.1 ([*Methylacidiphilum*]_D_) relative and absolute errors as a function of the known *Methylacidiphilum* ([*Methylacidiphilum*]_K_) concentrations in the model suspensions. (**b**) Derived *Galdieria* sp. RTK37.1 ([*Galdieria*]_D_) relative and absolute errors as a function of the known *Methylacidiphilum* ([*Methylacidiphilum*]_K_) concentrations in the model suspensions. Data points represent the relative or absolute error of the derived concentrations when compared to the known concentrations; error bars represent one standard deviation (*n* = 3). Positive errors represent that the estimated value is higher than the actual value. For each graph, *Methylacidiphilum* sp. RTK17.1 concentrations were varied while *Galdieria* sp. RTK37.1 concentrations were kept constant at the values shown. The subscript “D” stands for derived value, while “K” stands for known value. All concentration values represent optical densities (A.U.) measured at 600 nm. Errors for additional model suspensions are shown in Figure A5.

The relative and absolute errors of the derived concentrations were also analyzed (Figure 4). A summary of the findings can be seen in Table 1. For [*Galdieria*]_K_ ≤ 1.00 A.U., relative errors for both [*Galdieria*]_D_ and [*Methylacidiphilum*]_D_ remained < 10%, with absolute errors within ± 0.1 A.U. However, as [*Galdieria*]_K_ increased, so did relative and absolute errors. When [*Galdieria*]_K_ > 1.00 A.U., errors and standard deviations became more pronounced, particularly when [*Methylacidiphilum*]_D_ was < 0.5 A.U. at [*Galdieria*]_K_ OD_600_ = 3.23 A.U. Nevertheless, even at higher concentrations, most relative errors remained below 20%, and absolute errors were within ± 0.2 A.U. These trends align with the behavior predicted by Stokes’ law for ideal suspensions, where particles sediment independently at low concentrations [49]. As concentration increases, cells collide more frequently, bringing methanotrophs and microalgae into close contact. These interactions could promote adhesion between cells. Such interactions resemble bioflocculation, where the presence of bacteria can induce the rapid formation of multicellular aggregates in bacteria–microalgae cocultures [2]. Floc formation occurs when microorganisms come into contact and adhere to one another [50], often facilitated by exopolysaccharides (EPS), which neutralize cell surface charges and promote aggregation [51]. *Galdieria sulphuraria* can produce up to 115.8 mg L^−1^ of EPS [52], while EPS production in *Methylacidiphilum* spp. has not been documented. However, other methanotrophs, such as *Methylomicrobium alcaliphilum* 20z, can secrete up to 2.64 g L^−1^ of EPS [53]. Thus, it is plausible that EPS production occurs in *Methylacidiphilum* sp. RTK17.1 and *Galdieria* sp. RTK37.1 cocultures, contributing to the observed measurement errors. Floc formation is concentration-dependent [47], and aggregation efficiency is influenced by the bacterial–algal ratios [50]. Consequently, elevated *Galdieria* sp. RTK37.1 concentrations may, correspondingly, increase measurement errors. A similar phenomenon was reported in flow cytometric quantification of *Methylococcus capsulatus* and *Chlorella sorokiniana* cocultures [21], where methanotroph adhesion to microalgae led to measurement errors as high as 88.3% for *M*. *capsulatus* and 18.0% for *C*. *sorokiniana*. In contrast, this effect was not observed in cocultures involving species with similar cell sizes [21].

**Figure 5:**
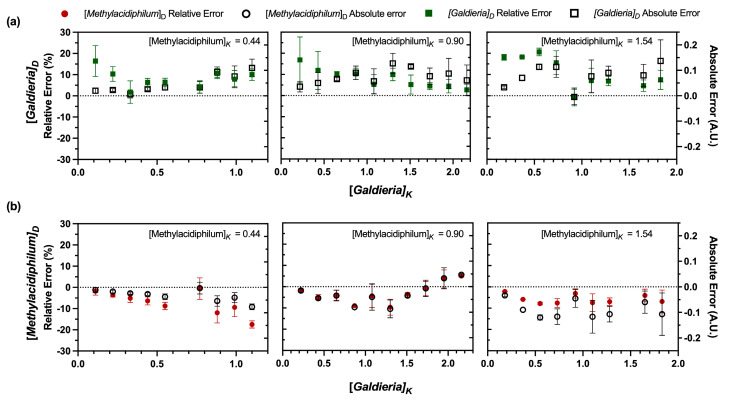
Relative and absolute errors of the derived concentrations of *Methylacidiphilum* sp. RTK17.1 and *Galdieria* sp. RTK37.1 in model suspensions using the DSOF method. (**a**) Derived *Galdieria* sp. RTK37.1 ([*Galdieria*]_D_) relative and absolute errors as a function of the known *Galdieria* ([*Galdieria*]_K_) concentrations in the model suspensions. (**b**) Derived *Methylacidiphilum* sp. RTK17.1 ([*Methylacidiphilum*]_D_) relative and absolute errors as a function of the known *Galdieria* ([*Galdieria*]_K_) concentrations in the model suspensions. Data points represent the relative or absolute error of the derived concentrations when compared to the known concentrations; error bars represent one standard deviation with *n* = 3. Positive errors represent that the estimated value is higher than the actual value. For each box, *Galdieria* sp. RTK37.1 concentrations were varied, and *Methylacidiphilum* sp. RTK17.1 remained constant at the concentration shown. The subscript “D” stands for derived value, while “K” stands for known value. All concentrations are optical densities, in absorbance units, measured at 600 nm.

The DSOF method tended to underestimate methanotroph concentrations and overestimate microalgal concentrations. This bias likely arises from the underlying assumption that *Methylacidiphilum* sp. RTK17.1 cells remain entirely in the supernatant after centrifugation. In practice, even at weak centrifugal forces, a fraction of methanotroph cells were removed (Figure A2). At dilute methanotroph concentrations, this loss was minimal and below the threshold of spectrophotometric determination. As concentrations increased, however, the same fractional loss presented as a larger absolute number of cells. Since the DSOF method assumed total OD_600_ as additive, underestimating methanotroph biomass entailed a corresponding inflation of microalgae biomass (Figure 4). The influence of increasing bacterial cell concentrations on the DSOF method was clearly demonstrated: at [*Methylacidiphilum*]ₖ = 1.54 A.U., deviations from known methanotroph concentrations remained within ± 0.1 A.U. (Figure 3b and Figure 5). These findings suggest that incorporating a concentration-dependent correction factor could improve DSOF accuracy for more concentrated suspensions by accounting for systematic methanotroph sedimentation. In contrast, *Galdieria* sp. RTK37.1 showed greater error at lower concentrations, primarily due to the unintended removal of methanotroph cells from the supernatant (Figure 5a). While this removal was relatively minor in absolute terms, it disproportionately affected estimates at low microalgal concentrations, resulting in higher relative error. A similar concentration-dependent bias has been reported in flow cytometry. For example, Badr et al. [21] observed deviations beginning at ≥0.015 g_DW_ L^−1^ for *M. alcaliphilum*, ≥0.08 g_DW_ L^−1^ for *Synechococcus* sp. PC7002, ≥0.04 g_DW_ L^−1^ for *M. capsulatus*, and ≥0.06 g_DW_ L^−1^ for *C. sorokiniana*. In comparison, the DSOF method showed deviations only at substantially greater concentrations (*Galdieria*: 0.308 g_DW_ L^−1^, OD_600_ = 1.00 A.U.; *Methylacidiphilum*: 0.222 g_DW_ L^−1^, OD_600_ = 0.50 A.U.). Nevertheless, most relative errors for *Galdieria* sp. RTK37.1 remained < 20%, and was typically under 10% for [*Galdieria*]ₖ ≥ 0.50 A.U. For *Methylacidiphilum*, errors were generally < 10%, with absolute errors falling between −0.2 and 0.05 A.U.

**Figure 6:**
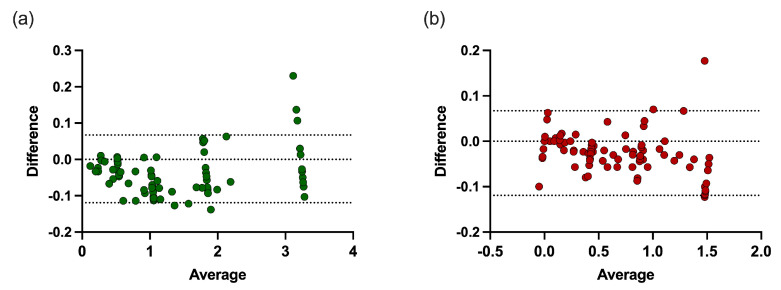
Bland–Altman analysis comparing DSOF-estimated and known concentrations for (**a**) *Galdieria* sp. RTK37.1 and (**b**) *Methylacidiphilum* sp. RTK17.1. Dashed lines represent the 95% limits of agreement. For *Methylacidiphilum*, most points fall within the limits, with no discernible trend (bias = 0.026, root mean squared error (RMSE) = 0.05). For *Galdieria*, values < 2.5 A.U. show good agreement (bias = −0.036, RMSE = 0.07), while higher biomass concentrations (>3.0 A.U.) display a tendency toward increasing variance.

To assess DSOF performance, we conducted Bland–Altman and root mean squared error (RMSE) analyses (Figure 6). For *Methylacidiphilum*, the mean bias was 0.026 A.U. with 95% limits of agreement between −0.067 and 0.119 A.U.; only one point fell outside these bounds, and no trend was observed, indicating strong agreement across concentrations. The RMSE of 0.05 A.U. confirmed low prediction error relative to the OD range. For *Galdieria*, the mean bias was −0.036 A.U. with limits between −0.155 and 0.182 A.U. Most values fell within these bounds, especially at OD < 2.5 A.U., where no trend was evident. At higher OD (~3.2 A.U.), deviations increased, and some points exceeded the limits, consistent with greater error under elevated biomass. Still, the RMSE of 0.07 A.U. shows errors remained modest compared with the overall range. Overall, DSOF demonstrated minimal bias and strong agreement with reference values. It performs best at moderate concentrations, with larger deviations only at very high biomass. Even under these conditions, absolute errors remained within ±0.2 A.U., supporting DSOF as a practical, low-cost tool for coculture monitoring.

Direct comparison of DSOF with other coculture quantification methods is limited by scarce error reporting. The E-C protocol [21] quantified biomass in *M. alcaliphilum*–*Synechococcus* and *M. capsulatus*–*C. sorokiniana* cocultures, showing strong agreement with flow cytometry (R^2^ = 0.90–0.98) and outperforming it at high concentrations. While E-C accuracy was unaffected by cell concentration within the tested ranges, it is unsuitable for *Methylacidiphilum-Galdieria* cocultures, as both species fix CO_2_ autotrophically [54], creating an unaccounted CO_2_ sink, and each can utilize alternative carbon sources [22,23,33,34,44,55,56], violating the mass-balance assumptions on which E-C relies.

### 3.2. Application of the DSOF Method to Monitor Growth Dynamics in Batch Cocultures

**Figure 7:**
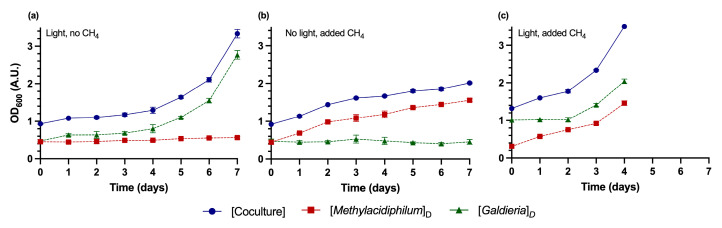
Growth of cocultures of *Methylacidiphilum* sp. RTK17.1 and *Galdieria* sp. RTK37.1. (**a**) Coculture with no added CH_4_ to suppress *Methylacidiphilum* growth. (**b**) Coculture with added CH_4_ but covered in foil to suppress *Galdieria* growth. (**c**) Coculture with added CH_4_ and no foil to allow growth of both microorganisms. Methanotroph and microalgae abundance in cocultures were calculated using the DSOF method. Cocultures were grown in V4 medium at 45 °C, 150 rpm, and with 40 μmol m^−2^s^−1^ warm white LED illumination in 1 L gastight bottles on a shaking incubator. Each day, headspace gas concentrations were replenished to avoid nutrient limitations. Cultures were grown in triplicate; error bars represent one standard deviation (*n* = 3).

Next, the DSOF method was applied to evaluate member dynamics in actively growing batch cocultures. For these experiments, three conditions were tested: (1) methanotroph growth suppression (no CH_4_), (2) microalgae growth suppression (no light), and (3) unrestricted growth of both microorganisms (Figure 7). As expected, in the absence of CH_4_, *Methylacidiphilum* sp. RTK17.1 concentrations did not increase significantly from an initial OD_600_ of 0.46 (±0.02 A.U., *p*-value > 0.05, Figure 7a). In contrast, *Galdieria* sp. RTK37.1 exhibited a three-day lag phase before growing from an initial OD_600_ of 0.48 (±0.04 A.U.) to a final concentration of 2.77 (±0.12 A.U., 577% increase, µ = 0.017 h^−1^). Conversely, in the absence of light, *Galdieria* growth was effectively suppressed, with OD_600_ values remaining stable (0.47 ± 0.06 A.U.). Meanwhile, *Methylacidiphilum* sp. RTK17.1 cell concentrations increased by 331% (µ = 0.0055 h^−1^), from an initial OD_600_ of 0.46 (±0.06 A.U.) to 1.56 (±0.04 A.U.).

The DSOF method was also successfully able to quantify the simultaneous growth of both *Methylacidiphilum* sp. RTK17.1 and *Galdieria* sp. RTK37.1 in coculture (Figure 7c). Following a two-day lag period consistent with previous reports [52,57,58], *Galdieria* sp. RTK37.1 biomass increased from 1.01 A.U. to 2.04 (± 0.06 A.U., µ = 0.015 h^−1^). Growth rates were not significantly different (*p* > 0.05) from those observed in the methanotroph-suppressed coculture (Figure 7a). Concomitantly, *Methylacidiphilum* sp. RTK17.1 concentrations increased from 0.31 to 1.46 A.U. (µ = 0.014 h^−1^), a rate 2.55x faster than in the microalgae-suppressed coculture. This finding aligns with previous observations that the oxygenic activity of *Galdieria* sp. RTK37.1 is able to enhance CH_4_ consumption of *Methylacidiphilum* sp. RTK17.1 in batch co-cultures [6]. When compared to axenic batch cultures, *Galdieria* growth rates were not significantly different (*p* > 0.05) from rates reported previously [6], while *Methylacidiphilum* growth rates were 12% faster (*p* < 0.05). This difference was likely due to the DSOF coculture experiments involving daily replenishment of O_2_, CO_2_, and CH_4_ to prevent substrate limitations. Collectively, these results indicate that the DSOF method was able to accurately and precisely quantify relative microalgae and methanotroph cell concentrations, with acceptable error, in actively growing cocultures.

### 3.3. Outlook and Limitations of the DSOF Method

Despite its speed, simplicity, and reliability, the DSOF method has limitations. It has not been validated under nitrogen-limiting conditions, where both *Galdieria* spp. and *Methylacidiphilum* spp. may accumulate glycogen [4,5,22,24], potentially increasing methanotroph cell size or density, accelerating sedimentation, and altering separation efficiency. Nitrogen-starved *Galdieria* also loses pigments, particularly phycocyanin [59], which may impair fluorescence-based measurements. These effects could substantially increase measurement error, requiring caution and possible adjustment of centrifugation parameters, though further validation is needed. The method was also only tested at total OD_600_ ≤ 5.0 A.U., with errors increasing at higher concentrations; introducing a concentration-dependent correction factor or diluting samples may mitigate this. A correction factor could be introduced into Equation (3) by incorporating a fractional sedimentation term for *Methylacidiphilum*. This factor could be modeled under different assumptions: (i) simple Stokes’ law behavior [47]; (ii) a Richardson–Zaki type relationship to capture biomass-dependent settling effects [47,49]; or (iii) empirical polynomial or logarithmic regression corrections, as commonly applied to OD-based biomass estimations [60,61,62]. The most appropriate model could then be calibrated using mixtures of *Methylacidiphilum* and *Galdieria* across a range of concentrations.

In this study, OD_600_ measurements were recorded relative to sterile cultivation medium as a blank. While OD_600_ provides a convenient and rapid proxy for biomass, it can also be influenced by extracellular metabolites or changes in medium composition over time, not exclusively by cell density [63,64]. In our defined media systems, such contributions appeared minimal; however, in natural or industrially derived feedstocks, suspended solids or metabolic byproducts could introduce additional scattering effects [64,65]. This limitation should be considered when applying DSOF in feedstocks containing suspended solids or with complex media, where background interferences may reduce measurement accuracy.

Because DSOF relies on differences in sedimentation rates and pigment fluorescence, it could potentially apply to other photoautotroph–methanotroph cocultures wherein large cell size differences exist. Thus, we speculate DSOF should be applicable to other microalgae–methanotroph cocultures, provided centrifugation parameters are optimized for maximum separation efficiency. However, minimal size differences, such as between *Synechococcus* spp. and *M. alcaliphilum* (Table A2), for example, may render DSOF unsuitable for most cyanobacteria–methanotroph pairs.

Our results demonstrate that the DSOF method provides a simple, rapid, and accurate tool for quantifying the relative abundance of thermoacidophilic microalgae and methanotrophs in coculture. Unlike conventional methods such as flow cytometry or qPCR, DSOF requires only standard laboratory equipment and minimal sample preparation, which makes it practical for routine use in experimental and applied settings.

Accurate differentiation of coculture members is particularly important because the performance of microalgae–bacteria systems depends strongly on inoculum proportion, biomass stability, and biochemical composition. This has been demonstrated in applied contexts such as wastewater treatment, where inoculation strategies influence biomass yield, protein/carbohydrate/lipid content, and photo-granule formation [14]. The DSOF method could therefore support optimization of consortia performance in applied biotechnologies where monitoring mixed populations remains a major challenge.

Monitoring cocultures typically relies on high-resource methods such as flow cytometry, molecular tools, or microscopy. However, as noted in recent reviews, physical and biological traits such as aggregation, sedimentation, and pigment variability make accurate quantification difficult with these approaches [30]. By exploiting differences in sedimentation and autofluorescence, DSOF offers a simpler alternative that addresses these challenges without requiring specialized infrastructure.

Our findings also align with recent spectroscopic approaches that combine optical density, autofluorescence, and UV–Vis measurements to improve estimation of algal biomass [43]. While such methods have been validated in monocultures, their application in cocultures remains limited due to overlapping optical signals. DSOF extends this principle by incorporating a physical separation step, thereby enabling accurate quantification in mixed systems where purely spectroscopic methods may fail.

Beyond experimental validation, the scalability of DSOF suggests potential application as a Process Analytical Technology (PAT) tool for real-time bioprocess monitoring. Recent advances in PAT for synthetic cocultures have highlighted the need for robust, low-cost tools to track biomass dynamics and metabolite fluxes in real time [26]. By integrating sedimentation-based separation with optical readouts, DSOF provides a complementary and accessible approach that could be implemented in routine monitoring and control strategies for cocultures, including microalgae–methanotroph systems.

## 4. Conclusions

In this study, a method based on differential sedimentation, optical density, and autofluorescence was developed and validated in a series of model (non-growing) and actively growing coculture experiments. Findings show the DSOF method can accurately measure *Galdieria* sp. RTK37.1 and *Methylacidiphilum* sp. RTK17.1 and abundance with <0.1 A.U. absolute error when [*Galdieria*]_K_ ≤ 2.0 A.U., and [*Methylacidiphilum*]_K_ ≤ 1.5 A.U. These errors increase to ±0.2 A.U., for 2.0 A.U. < [*Galdieria*]_K_ ≤ 3.23 A.U. Overall, the DSOF method tends to underestimate *Methylacidiphilum* concentrations, likely because the method assumes no methanotroph cells sediment during centrifugation. Despite its speed and simplicity, DSOF has not yet been validated under nitrogen-limiting conditions, where glycogen accumulation and pigment loss in *Galdieria* could impair accuracy, nor at higher biomass concentrations where scattering effects and concentration-dependent errors become more pronounced. Nevertheless, the method’s reliance on sedimentation and pigment fluorescence makes it potentially adaptable to other microalgae–methanotroph cocultures, provided that sufficient differences in cell size exist and centrifugation parameters are optimized for separation efficiency

Collectively, the results show that the DSOF method is an easy, quick, and accurate method to measure relative concentrations of *Methylacidiphilum* sp. RTK17.1 and *Galdieria* sp. RTK37.1, which does not depend on gas concentrations and only requires small volumes of liquid sample. This allows for the rapid and simple measuring of the relative concentration of microalgae and methanotrophs in coculture. Therefore, this method enables near-‘real-time’ monitoring of coculture response to different growth conditions, which should help to expand understanding of microalgae–methanotroph cocultures and interactions, and support their potential application in circular bioeconomy processes. While the method was developed for one algal-methanotroph pair, the conceptual framework, combining differential sedimentation with optical and fluorescence measurements, could be adapted to other systems where cell size or pigment differences exist, thereby providing a practical tool for bioprocess optimization

## Figures and Tables

**Table 1:** Summary of the relative and absolute errors of the derived concentrations of *Methylacidiphilum* sp. RTK17.1 and *Galdieria* sp. RTK 37.1 in artificial suspensions using the DSOF method.

Known Values (A.U.)		*Methylacidiphilum* sp. RTK17.1 Error		*Galdieria* sp. RTK37.1 Error	
RTK37.1 (A.U.)	RTK17.1 (A.U.)	Absolute (A.U.)	Relative (%)	Absolute (A.U.)	Relative (%)
<0.5	<0.5	±0.10	<10	±0.05	<20
<0.5	0.5–1.5	±0.10	<10	±0.10	<10
0.5–1.0	<1.0	±0.10	<10	±0.10	<10
0.5–1.0	1.0–1.5	±0.10	<10	±0.10	10–15
1.0–2.0	<0.5	±0.10	<10	±0.10	<10
1.0–2.0	0.5–1.0	±0.10	<10	±0.10	<10
1.0–2.0	1.0–1.5	±0.15	<10	±0.15	<10
2.0–3.2	<1.0	±0.10	<20	±0.10	<5
2.0–3.2	1.0–1.5	±0.20	<10	±0.15	<10

**Figure A5:**
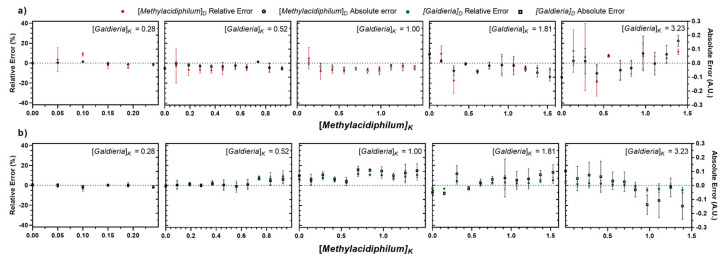
Expanded relative and absolute errors of the derived concentrations of *Methylacidiphilum* sp. RTK17.1 and *Galdieria* sp. RTK37.1 in model suspensions using the DSOF method for the constant *Galdieria* concentration groups. (**a**) Derived *Methylacidiphilum* sp. RTK17.1 ([*Methylacidiphilum*]_D_) relative and absolute errors as a function of the known *Methylacidiphilum* ([*Methylacidiphilum*]_K_) concentrations in the model suspensions. (**b**) Derived *Galdieria* sp. RTK37.1 ([*Galdieria*]_D_) relative and absolute errors as a function of the known *Methylacidiphilum* ([*Methylacidiphilum*]_K_) concentrations in the model suspensions. Data points represent the relative or absolute error of the derived concentrations when compared to the known concentrations; error bars represent one standard deviation (*n* = 3). For each graph, *Methylacidiphilum* sp. RTK17.1 concentrations were varied while *Galdieria* sp. RTK37.1 concentrations were kept constant at the values shown. All concentration values represent optical densities (A.U.) measured at 600 nm.

## Data Availability

Data is available upon request.

## Appendix A

**OD_600_ values are additive for *Methylacidiphilum* sp. RTK17.1 and *Galdieria* sp. RTK37.1**

To test whether *Methylacidiphilum* sp. RTK17.1 and *Galdieria* sp. RTK37.1 OD_600_ values were additive; model “coculture” suspensions were prepared from axenic stock suspensions across a range of predefined OD_600_ values and measured using a Ultrospec 10 cell density meter (Amersham Bioscience, United Kingdom). Measured suspension values were compared to “known” values (the summation of *Methylacidiphilum* sp. RTK17.1 and *Galdieria* sp. RTK37.1 individual OD_600_ values). Results are shown in Figure A1.

**There is no significant centrifugation of methanotrophic cells**

To test whether methanotroph cells were centrifuged to a relevant degree, axenic suspensions of *Methylacidiphilum* sp. RTK17.1 (OD_600_ = 0.88) and *Galdieria* sp. RTK37.1 (OD_600_ = 2.4) were centrifuged at 380 x g for variable periods of time using a microcentrifuge (Biofuge Pico, Heraeus Instruments). OD_600_ was measured from the suspensions and the supernatants using a Ultrospec 10 cell density meter (Amersham Bioscience, United Kingdom), and the fraction of supernatant retained biomass was then calculated. For times < 50 s, retained biomass for *Methylacidiphilum* RTK17.1 was >90%. Results are shown in Figure A2.

**The fraction of microalgae remaining in the supernatant can be approximated by the proportion of fluorescence of the supernatant and the original coculture**

To test whether fluorescence (emission 670 nm, excitation at 590 nm) was proportional to OD_600_ for *Galdieria* sp. RTK37.1, and that *Methylacidiphilum* sp. RTK17.1 exhibited no fluorescence at 670 nm when excited at 590 nm. Dilutions of stock suspensions were made. The suspensions’ OD_600_ were measured using an Ultrospec 10 cell density meter (Amersham Bioscience, United Kingdom), and the fluorescence (670 nm emission, 590 nm excitation) was measured using a microplate reader (Varioskan Lux, Thermo Scientific). *Galdieria* sp. RTK37.1 fluorescence was proportional to OD_600_, and *Methylacidiphilum* sp. RTK17.1 showed no fluorescence. Results are shown in Figure A3.

To test whether supernatant *Galdieria* sp. RTK37.1 fluorescence (670 nm emission, 590 nm excitation) was still proportional to OD_600_, *Galdieria* sp. RTK37.1 (OD_600_ = 2.4) suspensions were centrifuged at 380 x g for variable periods of time using a microcentrifuge (Biofuge Pico, Heraeus Instruments, Germany). Suspension and supernatant’s OD_600_ and fluorescence were measured, and the fraction of retained biomass and fluorescence was calculated. After centrifugation, *Galdieria* sp. RTK37.1 fluorescence was proportional to OD_600_.

**Table A1:** Initial CO_2_, CH_4_, and O_2_ headspace gas concentrations for DSOF evaluation in live coculture batch experiments. Uncertainties represent one standard deviation (*n* = 3).

Desired Condition	Headspace Concentrations (% *v*/*v*)		
	O_2_	CH_4_	CO_2_
*1. Methylacidiphilum* growth was suppressed	14.32 ± 0.01	0	17.29 ± 0.23
*2. Galdieria* growth suppressed	14.27 ± 0.05	10.00 ± 0.04	6.55 ± 0.07
*3. Galdieria* and *Methylacidiphilum* growth allowed	15.06 ± 0.09	6.94 ± 0.15	10.60 ± 0.04

**Table A2:** Sizes of photoautotrophs and methanotrophs in commonly studied cocultures.

Strain	Guild	Morphology & Size (l × w)	Reference
*Synechococcus* spp. *Methylomicrobium alcaliphilum*	phototroph methanotroph	coccus: 0.6–2.1 µm rod: 1.2–3.0 µm	[66,67]
*Chlorella sorokiniana* *Methylococcus capsulatus*	phototroph methanotroph	coccus: 2–5 µm coccus: 0.7–1.0 µm	[68,69]
*Galdieria* spp. *Methylacidiphilum* spp.	phototroph methanotroph	ovoid: 3.8–5.0 µm rod: 0.8–0.65 µm	[45,70]
